# The Protective Role of Mitochondrial Ferritin on Erastin-Induced Ferroptosis

**DOI:** 10.3389/fnagi.2016.00308

**Published:** 2016-12-20

**Authors:** Yue-Qi Wang, Shi-Yang Chang, Qiong Wu, Yu-Jing Gou, Linpei Jia, Yan-Mei Cui, Peng Yu, Zhen-Hua Shi, Wen-Shuang Wu, Guofen Gao, Yan-Zhong Chang

**Affiliations:** ^1^Laboratory of Molecular Iron Metabolism, College of Life Science, Hebei Normal UniversityShijiazhuang, China; ^2^Department of Nephrology, The Second Hospital of Jilin UniversityChangchun, China; ^3^Department of Clinical Laboratory, The Third Hospital of Hebei Medical UniversityShijiazhuang, China

**Keywords:** ferroptosis, iron, mitochondrial ferritin, ROS, LIP

## Abstract

Ferroptosis, a newly identified form of regulated cell death, is characterized by overwhelming iron-dependent accumulation of lethal lipid reactive oxygen species (ROS). Preventing cellular iron overload by reducing iron uptake and increasing iron storage may contribute to inhibit ferroptosis. Mitochondrial ferritin (FtMt) is an iron-storage protein that is located in the mitochondria, which has a significant role in modulating cellular iron metabolism. Recent studies showed that FtMt played inhibitory effects on oxidative stress-dependent neuronal cell damage. However, the potential role of FtMt in the progress of ferroptosis in neuronal cells has not been studied. To explore this, we established ferroptosis models of cell and drosophila by erastin treatment. We found that overexpression of FtMt in neuroblastoma SH-SY5Y cells significantly inhibited erastin-induced ferroptosis, which very likely was achieved by regulation of iron homeostasis. Upon erastin treatment, significant increases of cellular labile iron pool (LIP) and cytosolic ROS were observed in wild-type SH-SY5Y cells, but not in the FtMt-overexpressed cells. Consistent with that, the alterations of iron-related proteins in FtMt-overexpressed cells were different from that of the control cells. We further investigated the role of FtMt in erastin-induced ferroptosis in transgenic drosophila. We found that the wild-type drosophilas fed an erastin-containing diet didn't survive more than 3 weeks. In contrast, the FtMt overexpressing drosophilas fed the same diet were survival very well. These results indicated that FtMt played a protective role in erastin-induced ferroptosis.

## Introduction

The small molecule erastin triggers a unique iron-dependent form of non-apoptotic cell death in cancer cells and certain normal cells, which was recently identified as ferroptosis (Dixon et al., 2012). Ferroptosis is morphologically, biochemically and genetically distinct from apoptosis, necrosis and autophagy, as it is characterized by the overwhelming iron-dependent accumulation of lethal lipid reactive oxygen species (ROS) (Dixon et al., 2012; Xie et al., 2016). In ferroptosis, the generation of ROS involves nicotinamide adenine dinucleotide phosphate (NADPH)-dependent oxidase (NOX) family of superoxide-producing enzymes and lipid peroxidation. Ferroptosis is associated morphologically with the presence of shrunken, electron-dense mitochondria, thereby induced cell death (Dixon et al., 2012).

Iron plays a significant role in the progress of ferroptosis. Studies have shown that the ferroptosis process could be blocked by iron chelators and enhanced by exogenous lysosomal form of iron (Dixon et al., 2012; Eling et al., 2015). A study by Reed et al. found that knockdown the expression of iron regulator protein 2 (IRP2) significantly promoted erastin-induced killing (Reed and Pellecchia, 2012). IRP2 is an RNA-binding protein that controls the translation of a group of mRNAs involved in iron homeostasis. IRP2 expression decreases iron uptake by reducing transferrin receptor (TfR) expression and increases free iron storage by inducing ferritin expression (Henderson and Kühn, 1995; Schneider and Leibold, 2000; Meyron-Holtz et al., 2004). Thus, IRP2 plays a protective role in ferroptosis by preventing cellular iron overload. Reducing iron uptake and/or increasing iron storage may potentially inhibit ferroptosis.

Mitochondrial ferritin (FtMt) is an iron-storage protein located in mitochondria, and has been characterized structurally and functionally analogous to the well-characterized cytosolic H-ferritin (Levi et al., 2001). The expression of FtMt is restricted to mitochondria of cells of testes, the central nervous system, and some other high oxygen-consumption tissues (Santambrogio et al., 2007). FtMt has been shown to modulate cellular iron metabolism dramatically (Corsi et al., 2002; Drysdale et al., 2002; Cazzola et al., 2003). Previous studies suggested that overexpression of FtMt caused redistribution of iron from cytosol to mitochondria (Nie et al., 2005), thus high levels of FtMt in mitochondria may prevent cytosolic iron accumulation. Our previous studies have found that FtMt overexpression in neuroblastoma SH-SY5Y cell increased its resistance to oxidative stress (Shi et al., 2010; Wu et al., 2013), indicating FtMt is not only involved in storing cellular iron, but may also play a role in protecting mitochondria from iron-dependent oxidative damage (Yang et al., 2013; Gao and Chang, 2014). Our recent studies showed that FtMt overexpression protected 6-hydroxydopamine-indeced dopaminergic cell damage (You et al., 2016), and FtMt played inhibitory effects on neuronal tumor cell proliferation (Shi et al., 2015). Therefore, we speculate that the FtMt might also play a very important protective role in the new identified iron-dependent form of cell death, ferroptosis. We used an FtMt-overexpressed neuroblastoma SH-SY5Y cell line and induced ferroptosis by erastin treatment. In addition, an *in vivo* transgenic drosophila melanogaster model was also used. This study may provide inside into understanding the functions of FtMt in ferroptosis and developing new strategies for modulating ferroptosis-dependent cell death.

## Materials and methods

### Chemicals and antibodies

DMEM medium, fetal calf serum and HEPES were purchased from Gibco BRL (Grand Island, NY). Erastin, 2′,7′-dichlorofluorescein diacetate (DCF-DA), annexin V, propidium iodide (PI), 3-(4,5-dimethylthiazol-2-yl)-2,5-diphenyl-tetrazolium-bromide (MTT), Hoechst 33258, calcein-AM, penicillin and streptomycin were purchased from Sigma (St. Louis, MO). The salicylaldehyde isonicotinoyl hydrazone (SIH) was synthesized as described previously (Shi et al., 2010). The anti-mouse FtMt polyclonal antibody and anti-human FtMt monoclonal antibody were gifts from Prof. Sonia Levi. Anti-human β-actin polyclonal antibody (sc-130656, 1:3000 dilution), anti-human monoclonal L-ferritin antibody (sc-390558, 1:1000 dilution), anti-human VDAC2 polyclonal antibody (sc-32059, 1:1000 dilution) and anti-human NOX2 monoclonal antibody (sc-74514, 1:1000 dilution) were purchased from Santa Cruz Biotechnology (Santa Cruz, CA). Anti-human VDAC3 antibody (14451-1-AP, 1:500 dilution) was purchased from ProteinTech Group, Inc. (Chicago, IL). The anti-human polyclonal TfR1 antibody (PA5-27739, 1:2000 dilution) was purchased from Invitrogin (Shanghai, China).

### Cell lines and erastin treatment

The stable FtMt-expressing SH-SY5Y cell line (FtMt-SY5Y) and pcDNA3.1(−) empty vector transfected cell line (vector-SY5Y) were generated as described previously (Shi et al., 2010). Briefly, the amplified mouse FtMt cDNA, with a C-terminal hemagglutinin (HA) epitope sequence, was cloned into pcDNA3.1(−) vector to generate construct of FtMt-pcDNA3.1(−). The plasmids of FtMt-pcDNA3.1(−) and empty vector were transfected into SH-SY5Y cells, and stable transfectants were selected. The expression of mouse FtMt protein was confirmed with western blotting by using anti-HA antibody (Shi et al., 2010). The cells were maintained in DMEM supplemented with heat-inactivated fetal calf serum (10%, vol/vol), glucose (4.5 mg/ml), penicillin (100 U/ml) and streptomycin (100 μg/ml) in humidified 5% CO_2_ at 37°C. Erastin was dissolved in DMSO. The stock solution of erastin was prepared and stored at −20°C in the dark.

### Drosophila and erastin treatment

Three types of drosophila melanogasters wild-type (WT) W1118, elav-Gal4 and UAS-Fer3HCH were gifts from Prof. Bing Zhou at Tsinghua University. The USA-Fer3HCH flies overexpress Fer3HCH (a mitochondrial ferritin subunit) under control of the Gal4–upstream activating sequence (USA) system (Missirlis et al., 2006). The elav-Gal4 is a pan-neuronal expressing driver stain, which contributes to USA transgene expression in salivary glands, neuroectodermal cells and embryonic nervous system in early drosophila embryos (Tzortzopoulos and Skoulakis, 2007). To achieve overexpression of FtMt, we crossed the USA-Fer3HCH flies to elav-Gal4 flies; while the crossing of USA-Fer3HCH flies to W1118 (a strain not expressing Gal4) was used as control. The flies were cultured at 25 ± 3°C and 70–80% relative humidity, and fed on a standard corn flour-agar diet with yeast granules as the protein source. New hatching drosophilas were subjected to CO_2_ narcosis, and female and male drosophilas were separated under stereology microscope. For erastin resistance test, 3-day-old male flies (both WT and FtMt overexpressing strains) were chronically exposed to 10 μM erastin in diet, and the medium were changed every 3 days to avoid desiccation. Lethality of the flies was monitored until day 22. The Life span curves were obtained from independent trials with a minimum of 100 flies per experiment. Experimental data were presented as percentage of survival.

### Cell viability assay

Cell viability was measured by MTT assay according to the literature (Lu et al., 2009). In brief, the exponentially growing SH-SY5Y cells, FtMt-SY5Y cells and vector-SY5Y cells were seeded in a 96-well plate at a density of 1 × 10^4^ cells/well. After overnight incubation, the medium was replaced with fresh medium with or without 10 μM erastin, and cells were incubated for the subsequent 24 h. Erastin-treated or untreated cells were incubated in MTT (0.5 mg/mL) for another 4 h, and then cells were lysed with DMSO. The absorbance at 595 nm was measured with a Bio-Rad model 3550 microplate reader (Richmond, CA). The samples were measured in eight replicates, and each experiment was repeated three times.

### Measurement of intracellular ROS

The level of intracellular ROS was quantified by measuring the fluorescence of DCF-DA, according to Guo et al. (2005). After treatment with or without 10 μM erastin for 24 h, the cells were collected and washed 3 times with 1 × PBS, followed by incubation with 5 μM DCF-DA for 45 min at 37°C in the dark. Cells were then washed 3 times with 1 × PBS and resuspended in a buffer containing 130 mM NaCl, 5.4 mM KCl, 0.8 mM MgCl_2_, 1.8 mM CaCl_2_, 15 mM glucose and 5 mM HEPES, pH 7.4. The relative levels of fluorescence were quantified by fluorescence spectrophotometer (Synergy H4; 488 nm excitation and 525 nm emission).

### Measurement of mitochondrial ROS

All solutions and equipments for mitochondrial extraction were pre-cooled and kept at 0–4°C. Cells were centrifuged at 1000 g for 15 min, resuspended in ice-cold 500 μl of CHM buffer (150 mM MgCl_2_, 10 mM KCl, 25 mM Tris-HCl, PH 6.7 and 1 mM EDTA), and kept on ice for 2 min. Cells were homogenized with syringe by pipetting 20–30 times. 200 μl of ice-cold CHM containing 1M sucrose (final 0.25 M) was added and gently mixed with cells by repeated inversion. Nuclei were sediment by centrifugation at 1000 g, 4°C for 10 min, followed by another centrifugation after removing the supernatant at 10000 g, 4°C for 10 min. The pellet cell then washed with 1 mL sucrose/Mg^2+^ medium and centrifuged at 10000 g, 4°C for 10 min. The obtained mitochondria were resuspended with mt suspension medium I (0.25 M sucrose, 25 mM Tris-base, PH 7.0), and then plated in 96-well plates. The level of mitochondrial ROS was quantified by measuring the fluorescence of DCF-DA (Guo et al., 2005). The following procedures were same as the measurement of intracellular ROS.

### Measurement of intracellular labile iron pool (LIP)

The LIP levels were measured according to the literature (Epsztejn et al., 1997), with minor modifications. In brief, the exponentially growing SH-SY5Y cells, vector-SY5Y, and FtMt-SY5Y cells seeded in a 96-well plate at a density of 1 × 10^4^ cells/well. After overnight incubation, the medium was replaced with fresh medium with or without 10 μM erastin, and cells were incubated for another 24 h. After the cells were harvested, washed and resuspended in a buffer containing 140 mM NaCl, 5 mM KCl, 1 mM MgCl_2_, 5.6 mM glucose, 1.5 mM CaCl_2_, and 20 mM HEPES (pH 7.4). Calcein-AM was added at a final concentration of 0.25 mM. The reaction mixture was incubated for 30 min at 37°C, and then washed for 3 times. The cells were resuspended in their respective well. The fluorescence intensity of calcein-AM was quantified by Microplate reader (Synergy H4; 488 nm excitation and 525 nm emission). After the baseline was stable, SIH (final concentration of 100 μM) was added. The reaction mixture was subsequently incubated for 30 min at 37°C, and the fluorescence intensity was detected. The increase in fluorescence intensity reflected the levels of calcein-bound iron.

### Western blotting

The method of western blotting has been described previously by Shi et al. (2010). Briefly, cells were harvested, homogenized and lysed with lysis buffer (50 mM Tris–HCl, 150 mM NaCl, 0.02% NaN_3_, 100 μg/ml PMSF, 1 μg/ml aprotinin, 1 μg/ml pepstatin A, 2 μg/ml leupeptin and 1% Triton X-100). After centrifugation at 12,000 g for 30 min at 4°C, the supernatant was collected. Protein concentration was determined by a BCA Protein Assay kit (Pierce Biotechnology). ~40 μg of protein for each sample was resolved by 10% sodium dodecyl sulfate-polyacrylamide gel electrophoresis (SDS-PAGE) and then transferred to a nitrocellulose membrane. The membrane was blocked by incubating with 5% nonfat milk in PBS containing 0.1% Tween-20 (PBS-T) for 1 h, and then hybridized with primary antibodies overnight with constant agitation at 4°C. After washing 3 times for 15 min each with PBS-T, the membrane was incubated for 1 h with peroxidase-coupled secondary antibodies, and detected with the ECL plus Western Blotting Detection Reagents (Pierce Biotechnology, Rockford IL).

### Statistical analysis

All experiments were performed at least three times. One-way ANOVA was used to estimate the overall significance determined by Tukey's tests corrected for multiple comparisons. Data are presented as mean ± SD. A probability level of 5% (*p* < 0.05) was considered to be significant.

## Results

### FtMt expression significantly rescues erastin-induced neuronal cell death

**Figure 1:**
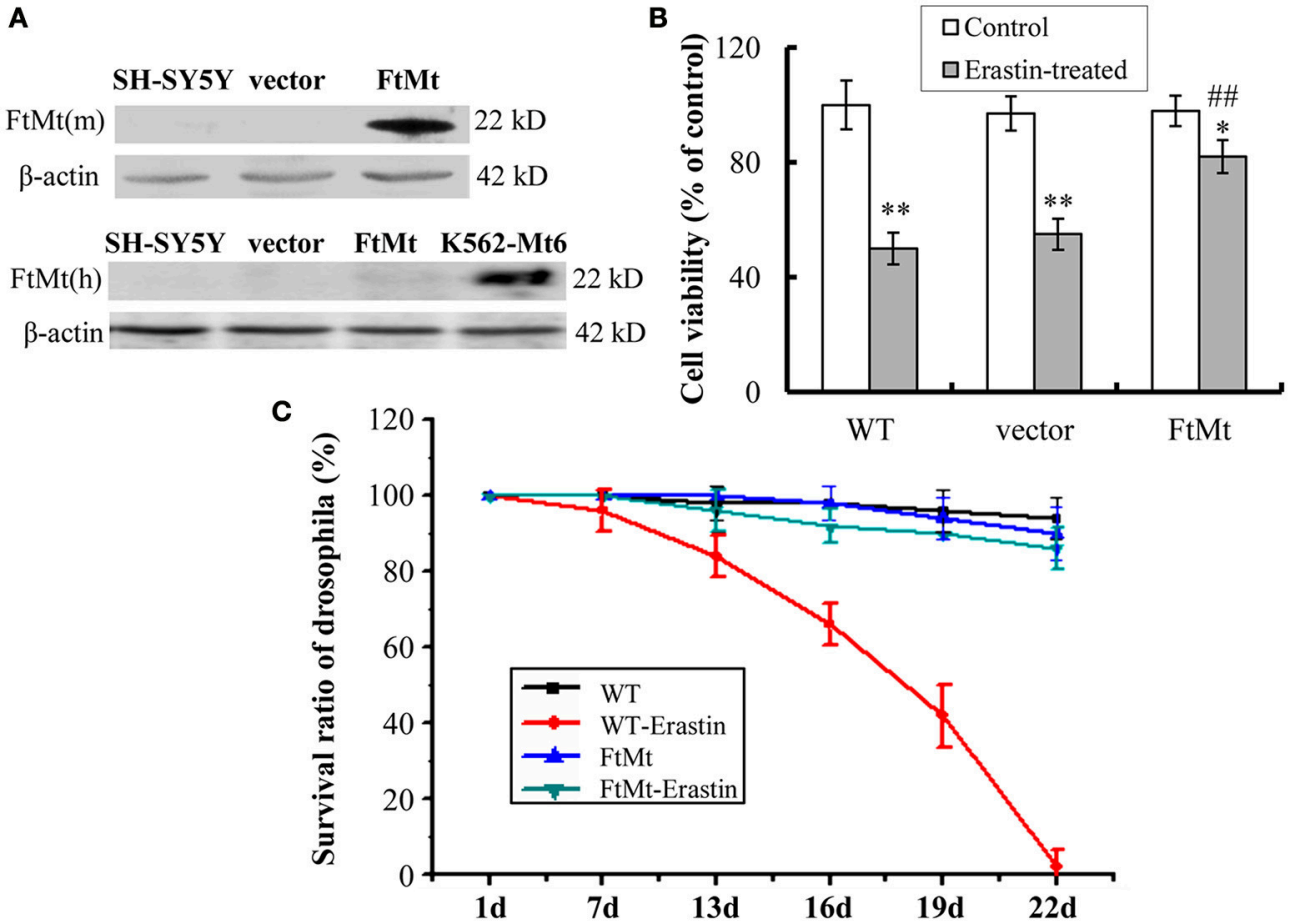
**Effects of FtMt on cell viability and survival rate of drosophilas treated with erastin. (A)** The expressions of exogenous mouse FtMt (top panel) and endogenous human FtMt (bottom panel) in FtMt overexpressed transfectants were examined by western blot using anti-mouse and anti-human FtMt antibodies, respectively. K562-Mt6 is a human FtMt overexpressing cell line (a gift from Prof. Sonia Levi), which is used as a positive control. **(B)** The cell viability was measured with the MTT assay. The wild-type (WT) SH-SY5Y cells, empty vector transfectants (vector), and FtMt overexpressed transfectants (FtMt) were treated with or without 10 μM erastin for 24 h. Data were presented as mean percentages of the cell viability compared with untreated WT control cells ± SD, *n* = 6 (^*^p < 0.05 and ^**^p < 0.01 vs. the untreated cells of same genotype; ##*p* < 0.01 vs. the erastin-treated vector control cells). **(C)** Modulatory effect of FtMt on erastin-induced mortality. Survival rates of the WT W1118 drosophilas and FtMt overpressing flies, exposed to 10 μM erastin containing or non-containing diet, were shown. Values are means ± SD (*n* = 100 flies/replicate; three replications/group; from three different experiments).

The overexpression of mouse FtMt in SH-SY5Y cells was confirmed by western blotting (Figure 1A). The endogenous expression of human FtMt in SH-SY5Y cell line was very low. To investigate the potential role of FtMt in erastin-induced ferroptosis, we treated FtMt overexpressing cells (FtMt-SY5Y), control SH-SY5Y and vector-SY5Y cells with erastin (10 μM) for 24 h. As shown in Figure 1B, remarkable decreases in the viability of SH-SY5Y and vector-SY5Y cells were observed. The viability of FtMt-SY5Y cells also decreased. However, the decrease is significantly less than that of the erastin-treated SH-SY5Y and vector-SY5Y cells, indicating that FtMt overexpression rescued erastin-induced neuronal cell death.

### FtMt overexpression protected erastin-induced death in drosophila

To obtain more comprehensive results, we studied the protective role of FtMt overexpression on ferroptosis *in vivo*. Transgenic FtMt overexpressing drosophilas were fed on a 10 μM erastin containing diet or non-containing diet, and their survival rates were monitored over a period of time. The WT drosophila under the same treatment condition was used as control. As shown in Figure 1C, more than 50% of mortality was observed in the WT drosophilas after 19 days of erastin treatment, and no drosophila survived longer than 22 days. Interestingly, the FtMt overexpressing drosophilas exposed to erastin-containing diet survived very well. Only about 12.5% of drosophilas died over the 22 days. These results implicated a protective role that FtMt played against erastin-induced ferroptosis *in vivo*.

### FtMt attenuates the increase of VDAC2/3 and NOX2 in erastin treatment

**Figure 2:**
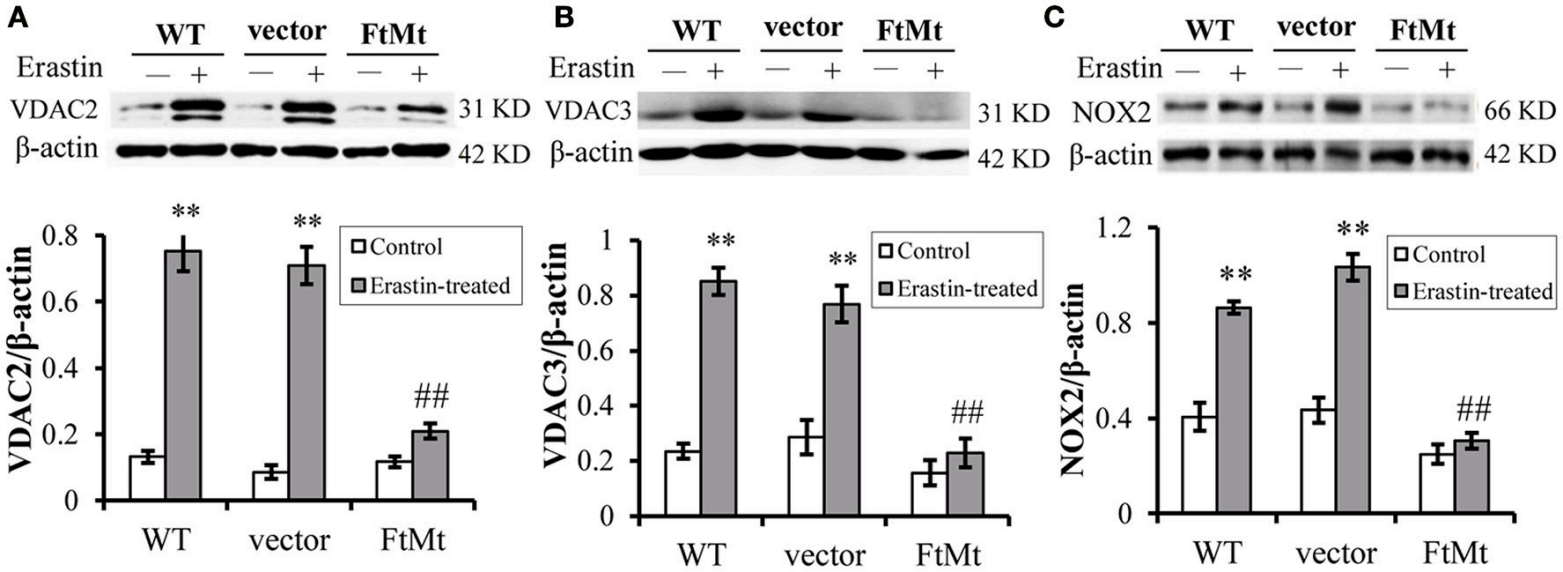
**FtMt attenuated the increase of VDAC2/3 and inhibited NOX2 activation under erastin treatment. VDAC2 (A)**, VDAC3 **(B)** and NOX2 **(C)** levels were determined by western blots. A representative blot image for each protein and its respective β-actin was shown. The expression levels in different groups were calculated by normalizing the specific bands to their respective β-actin bands, and presented as means ± SD, *n* = 6 (^**^p < 0.01 vs. the untreated cells of same genotype; ##*p* < 0.01 vs. the erastin-treated vector controls).

The voltage-dependent anion channels (VDACs), particularly VDAC2 and VDAC3, on mitochondrial membrane were found as the essential targets of erastin (Yagoda et al., 2007), and study by Dixon et al. had validated the upregulation of VDAC2 and VDAC3 with erastin treatment (Dixon et al., 2012). Therefore, we assessed the protein levels of VDAC2 and VDAC3 to further confirm the protective effect of FtMt on erastin treatment. We found that both the levels of VDAC2 and VDAC3 expression increased significantly in the SH-SY5Y and vector-SY5Y cells following erastin treatment (Figures 2A,B, respectively). However, the VDAC2 and VDAC3 levels didn't alter significantly in FtMt-SY5Y cells with erastin treatment as compared to the untreated FtMt-SY5Y cells, suggesting a specific protective effect of FtMt on erastin-induced ferroptosis.

Besides, we determined the activation of NADPH oxidase 2 (NOX2) in the three cell lines with or without erastin treatment. It has been suggested that NADPH oxidase (NOX1 to 5) could be critical in the production of ROS in ferroptosis (Dixon et al., 2012), and NOX2 appeared to be a major source of pathological oxidative stress in the central nervous system (Infanger et al., 2006; Sorce and Krause, 2009; Hur et al., 2010; Nayernia et al., 2014). Studies have shown that the activation of NOX2 enzyme remarkably promoted cell death in central nervous system diseases (Sorce and Krause, 2009; Hur et al., 2010; Nayernia et al., 2014; Marrali et al., 2016). Here, we found that, after erastin treatment, the NOX2 indeed expressed to a higher level after erastin treatment (Figure 2C), in both WT SH-SY5Y and vector-transfected control cells. The increases were statistically significant. Interestingly, the NOX2 level didn't increase much in the erastin-treated FtMt overexpressing cells (Figure 2C). This inferred that the protective role of FtMt in ferroptosis may attribute to its inhibitory effect on ROS production.

### FtMt expression attenuates erastin-induced accumulation of cytoplasmic ROS

**Figure 3:**
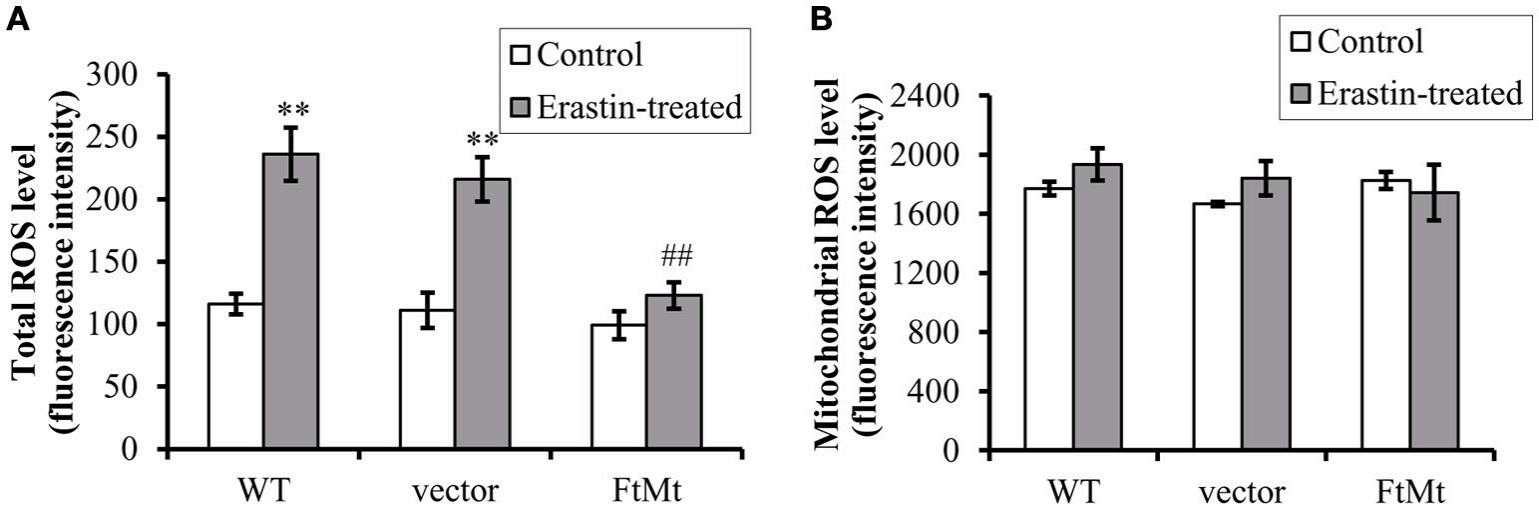
**FtMt expression attenuated erastin-induced accumulation of cytoplasmic ROS. (A)** Cells treated with or without 10 μM erastin were detected for ROS production by DCF-DA fluorescence. **(B)** The mitochondrial ROS was detected by DCF-DA. The fluorescence level for each group was presented as mean ± SD, *n* = 3 (^**^P < 0.01 vs. the untreated cells of same genotype; ##*p* < 0.01 vs. the erastin-treated vector controls).

Since erastin-induced ferroptosis has been shown to be closely associated with cytoplasmic ROS production, and the alterations of NOX2 were different among different cells, we therefore measured the ROS production both in cytoplasm and in mitochondria. As we predicted, erastin treatment led to substantial increases in intracellular ROS production in SH-SY5Y and vector-SY5Y cells (Figure 3A, *p* < 0.01). However, only a slight increase was observed in FtMt-overexpressed FtMt-SY5Y cells treated by erastin, which was not statistically significant. These inferred that overexpression of FtMt attenuated the erastin-induced accumulation of intracellular ROS. We further isolated mitochondria and detected mitochondrial ROS production. We found that the mitochondrial ROS production had no obvious difference with or without erastin treatment in all the three groups (Figure 3B). These were consistent with the previous reported features of ferroptosis.

### Effects of FtMt on the LIP level and iron metabolism under erastin treatment

**Figure 4:**
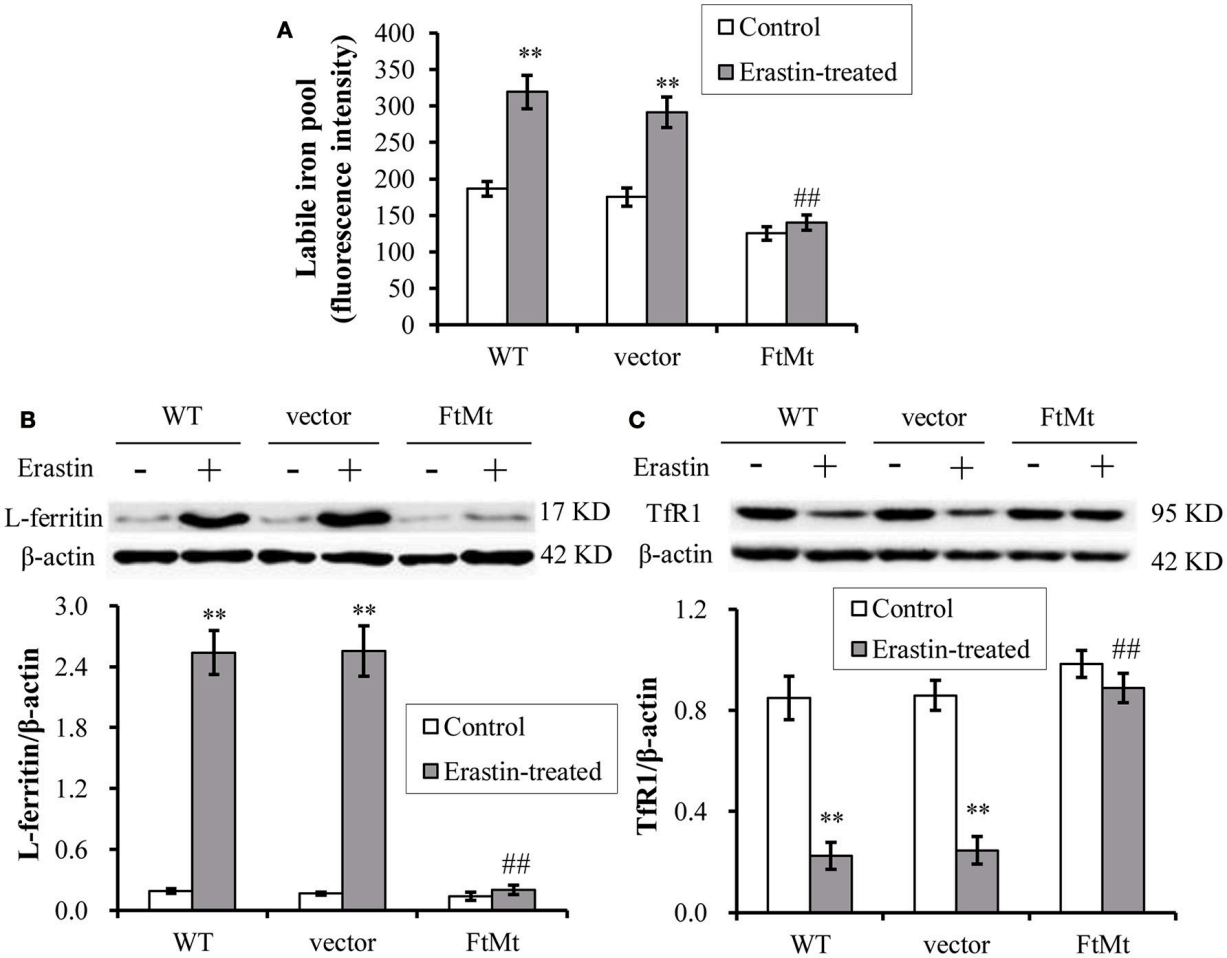
**Effects of FtMt on the LIP level and iron metabolism under erastin treatment. (A)** LIP levels were determined by the quenching of calcein-AM fluorescence method using fluorescence spectrophotometer. The LIP level was presented as mean ± SD; *n* = 3 (^**^p < 0.01 vs. the untreated cells of same genotype; ##*p* < 0.01 vs. the erastin-treated vector controls). Ferritin **(B)** and TfR1 **(C)** levels were determined by western blots. A representative blot image for each protein and its respective β-actin was shown. The expression levels in different groups were calculated by normalizing the specific bands to their respective β-actin bands, and presented as means ± SD, *n* = 6 (^**^p < 0.01 vs. the untreated cells of same genotype; ##*p* < 0.01 vs. the erastin-treated vector controls).

The intracellular labile iron pool (LIP) of mammalian cells has been suggested to be the cellular free-iron source that was relatively accessible for Fenton reaction. Researchers have shown that FtMt overexpression mobilized free iron and dramatically redistributed cytosolic iron into mitochondria (Corsi et al., 2002; Nie et al., 2005). The erastin-induced ferroptosis was also closely dependent on the intracellular iron level. Therefore, we determined the LIP level by using calcein-AM assay in the FtMt -SY5Y cells and control cells under erastin treatment. As shown in Figure 4A, LIP level in the control SH-SY5Y and vector-SY5Y cells increased significantly after erastin treatment, attesting the proposed damage effects of iron-dependent accumulation of ROS in ferroptosis. In contrast, the FtMt-SY5Y cells only showed a slight, insignificant increase in the LIP level after erastin treatment.

We then detected the expression levels of iron-related proteins, including iron-storage protein L-ferritin and iron-uptake protein transferrin receptor 1 (TfR1). We found the L-ferritin level dramatically upregulated in erastin-treated SH-SY5Y and vector-SY5Y cells, reaching more than 8-times that of the untreated control levels (Figure 4B). Again, the FtMt-SY5Y cells didn't show a large increase in the L-ferritin level following erastin treatment. In erastin-treated SH-SY5Y and vector-SY5Y cell, we also found the iron uptake protein TfR1 was significantly down-regulated (Figure 4C), suggesting the existence of a cellular regulation of iron metabolism, possibly by IRP, in response to the dramatically elevated intracellular iron level.

## Discussion

Ferroptosis is closely associated with the level of intracellular iron. The process of ferroptosis requires iron-dependent production of ROS generated by Fenton-type reactions (Dixon et al., 2012). Studies have shown that the ferroptosis process could be blocked by iron chelators, while it was enhanced by exogenous lysosomal form of iron (Dixon et al., 2012; Eling et al., 2015). Mitochondrial ferritin (FtMt), an iron storage protein located in mitochondria, was found in tissues with high levels of oxygen consumption (Santambrogio et al., 2007). FtMt protected mitochondria from iron-dependent oxidative damage by reducing cellular oxidative stress and ROS (Shi et al., 2010; Wu et al., 2013). In this study, we found that the cell viability markedly decreased in SH-SY5Y and vector-SY5Y cells after erastin treatment, but not in the FtMt overexpressing cells. This indicated that overexpression of FtMt protected neuroblastma SH-SY5Y cells from erastin-induced ferroptosis. In a transgenic drosophila model, the erastin-induced death in drosophila was suppressed by the overexpressed FtMt, which also indicated that FtMt indeed can suppress the erastin-induced ferroptosis.

In erastin-induced ferroptosis, the VDAC2 and VDAC3, present in the mitochondrial outer membrane, are the direct targets of erastin (Yagoda et al., 2007), and RNAi interference knockdown of VDAC2 may cause the resistance of erastin. VDAC is a monomeric, voltage-gated channel that allows passage of metabolites such as ATP, ADP, phosphocreatine and small ions across the mitochondrial outer membrane (Colombini, 2007; Young et al., 2007). In addition to its role in energy metabolism, VDAC has been tied to basic cellular processes such as cytochrome-c dependent apoptosis (Shimizu et al., 1999; Cheng et al., 2003). Our study found that FtMt can inhibit the erastin-induced elevation in VDAC2 and VDAC3 expression, which then resulted in the resistance of FtMt overexpressing cells to erastin damage. However, the protective mechanisms of FtMt on ferroptosis could be very complicated.

Similarly, the FtMt expression protected the cell from ferroptosis by inhibiting erastin-induced elevation of NOX2 levels. As a major NADPH oxidase isoform in generation of ROS, NOX2 activation remarkably promoted pathological oxidative stress in the central nervous system (Sorce and Krause, 2009; Nayernia et al., 2014). Up-regulation of NOX2 has been reported in the pathogenesis of Parkinson's disease in human patients and animal models (Glass et al., 2010), and NOX2 enzyme activation was also found in patients with chronic inflammatory demyelinating polyneuropathy (Marrali et al., 2016). Our findings indicated that NOX2-dependent oxidative stress participated in the erastin-induced SH-SY5Y cell death, whereas overexpression of FtMt suppressed the activation of NOX2.

Subsequently, we determined the intracellular and mitochondrial ROS levels in different cells with or without erastin treatment. Results showed that the intracellular ROS increased, but not the mitochondrial ROS, in the control SH-SY5Y cells; whereas FtMt overexpression significantly inhibited the erastin-induced increases in cytoplasmic ROS. Since the primary function of FtMt is iron-storage, and it has been shown to be involved in iron redistribution between cytosol and mitochondria (Nie et al., 2005), intracellular LIP level was detected in different cells. Consistent with previous reports, the LIP levels were dramatically elevated upon erastin treatment in control SH-SY5Y cells. However, FtMt significantly inhibited LIP increase induced by erastin, indicating that the antioxidative mechamism of FtMt against erastin may predominately carry out through regulating iron homeostasis. When iron levels elevated in the body, a cellular iron regulatory pathway, the IRE/IRP system, would be activated in order to attenuate the iron overload status. The expression of iron-storage protein ferritin will be up-regulated and iron-uptake protein TfR1 will be down-regulated. In our study, increased L-ferritin level and decreased TfR1 expression were observed in erastin-treated SH-SY5Y cells, whereas the FtMt overexpression diminished these alterations. These further attested the important role of FtMt in the modulation of iron metabolism, and suggested that the neuroprotective role of FtMt should be achieved through regulating iron homeostasis.

**Figure 5:**
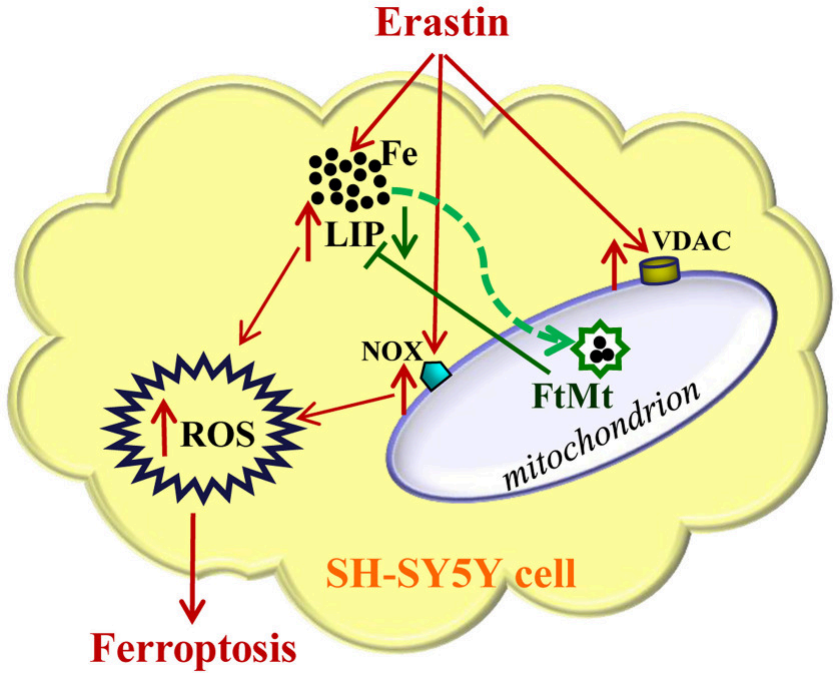
**A schematic representation of the proposed neuroprotective mechanism of FtMt on erastin-induced neuronal ferroptosis**. Extracellular erastin treatment induced ferropotosis in neuroblastoma SH-SY5Y cells, which involved the elevation of VDAC and NOX levels and also depended on the increase of LIP level. The free iron may donate electrons for the generation of ROS, and then cell is triggered to begin the process of ferroptosis. The overexpressed FtMt may withdraw iron from cytosol and inhibit ROS production. This in turn attenuated erastin-induced ferrotptosis. The damaging effects caused by erastin treatment were indicated with red arrows, while the protective effects of FtMt were indicated by green arrows.

In summary, our study suggested that FtMt played a significant protective role in erastin-induced ferroptosis (Figure 5). FtMt incorporated iron in mitochondria and directly inhibited the erastin-induced LIP elevation and ROS production in SH-SY5Y cells. The protection of FtMt on erroptosis was also reflected by the suppression of VDAC2/3 expression and NOX2 activation induced by erastin. This may link to its role in regulating iron homeostasis although the exact mechanisms from incorporating iron to altering the expressions of related genes could be very complicated. Our study revealed the protective effects of FtMt on erastin-induced ferroptotisis, which may provide insight into identifying new targets for inhibiting ferroptosis in neuronal cells.

## Author contributions

YW and SC: Performed most of the experiments, and contributed equally to this work. YG, LJ, QW, and YMC: Performed a small part of the experiments; PY, ZS, and WW: Participated in the design of the work and discussion of the results. YZC and GG: Conceived the work and revised the manuscript; GG and YW: Drafted the manuscript. All authors read and approved the final manuscript.

## Funding

This work was supported by the National Natural Science Foundation of China [grant numbers 31520103908, 31300898]; and the Natural Science Foundation of Hebei Province [grant number C2015205206].

### Conflict of interest statement

The authors declare that the research was conducted in the absence of any commercial or financial relationships that could be construed as a potential conflict of interest.

## Abbreviations

FtMt: Mitochondrial ferritin
LIP: cellular labile iron pool
ROS: reactive oxygen species
NADPH: nicotinamide adenine dinucleotide phosphate
NOX: NADPH-dependent oxidase
IRP2: iron regulator protein 2
TfR1: transferrin receptor 1
VDAC: voltage-dependent anion channels.
